# Supplementary material for: Targeting Mediator Kinase Cyclin-Dependent Kinases 8/19 Potentiates Chemotherapeutic Responses, Reverses Tumor Growth, and Prolongs Survival from Ovarian Clear Cell Carcinoma
Source: Cancers (Basel). 2025 Mar 10;17(6):941. doi: 10.3390/cancers17060941 (PMC11940259; doi:10.3390/cancers17060941)
Supplement: Supplementary file 1 [file cancers-17-00941-s001.zip › cancers-3467353-supplementary.pdf]

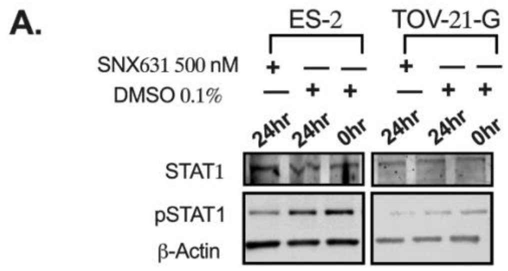

Supp Figure S1: Phosphorylation of STAT1 is inhibited with a subtherapeutic concentration of SNX631. Western blots of indicated proteins in ES-2 (ARID1A WT) and TOV-21-G (ARID1A mutated) upon treatment with 500nM SNX631 or vehicle (DMSO) control.

	SNX631 (μM)	Carboplatin (μM)	Carboplatin (μM) + SNX631 (nM)	Cisplatin (μM)	Cisplatin (μM) + SNX631 (nM)	Taxol (nM)	Taxol (nM) + SNX631 (nM)
**ES-2**	2.39	12.2	4.1 *(2.97)*	4.2	1.1 *(3.82)*	6.8	2.9 *(2.34)*
**TOV-21-G**	1.41	95.5	70.3 *(1.36)*	22.9	13.5 *(1.7)*	15.2	10.5 *(1.45)*
**RMG-1**	3.28	22.4	10.7 *(2.09)*	7	5.2 *(1.35)*	4.3	2.5 *(1.72)*
**SKOV-3**	6.14	210.3	72.3 *(2.91)*	29.6	14.2 *(2.08)*	3.4	2.7 *(1.26)*
**SKOV-3 CDK8/19 KO**	Undefined	20.7	14.7 *(1.41)*	3.7	3.1 *(1.19)*	2	1.6 *(1.25)*

Supp Figure S2: IC50 values for SNX631 combinations in seven-day growth inhibition assays with fold-change difference. IC50 values for drug combinations are shown for each drug combination and OC cell line. Fold-change decrease in IC50 relative to monotherapy treatment is presented in parentheses.

A.

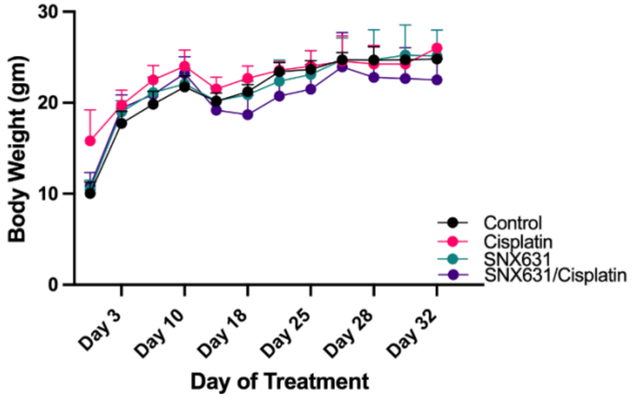

B.

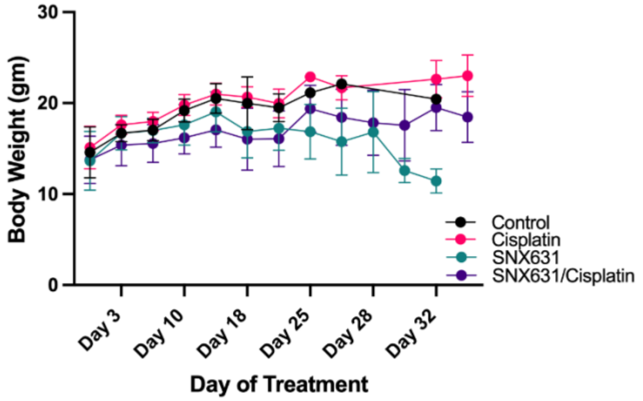

Supp Figure S3: Animal body weight over time in indicated groups in ES2-luc NU/J xenografts injected (A) subcutaneously or (B) intraperitoneally.

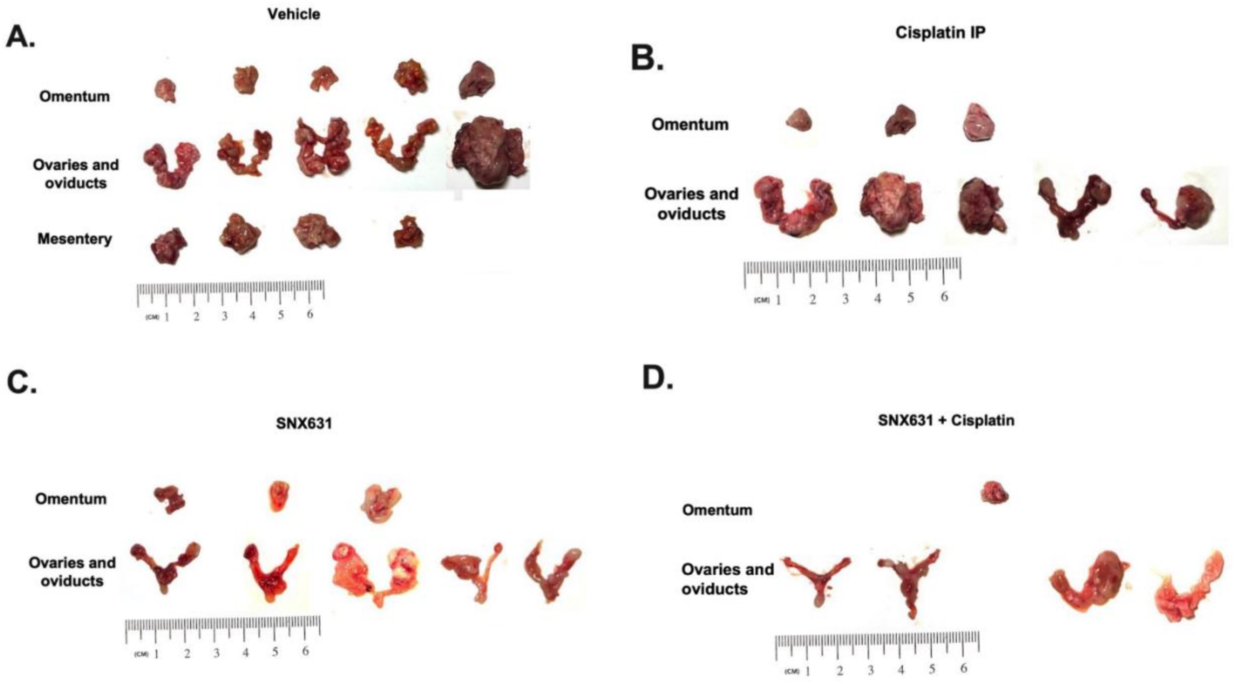

Supp Figure S4: Representative images of intraperitoneally injected tumors from the omentum, ovaries/oviducts, and mesentery for the four treatment groups at the endpoint: (A) vehicle, (B) cisplatin, (C) SNX631, and (D) SNX631 + cisplatin.

A.

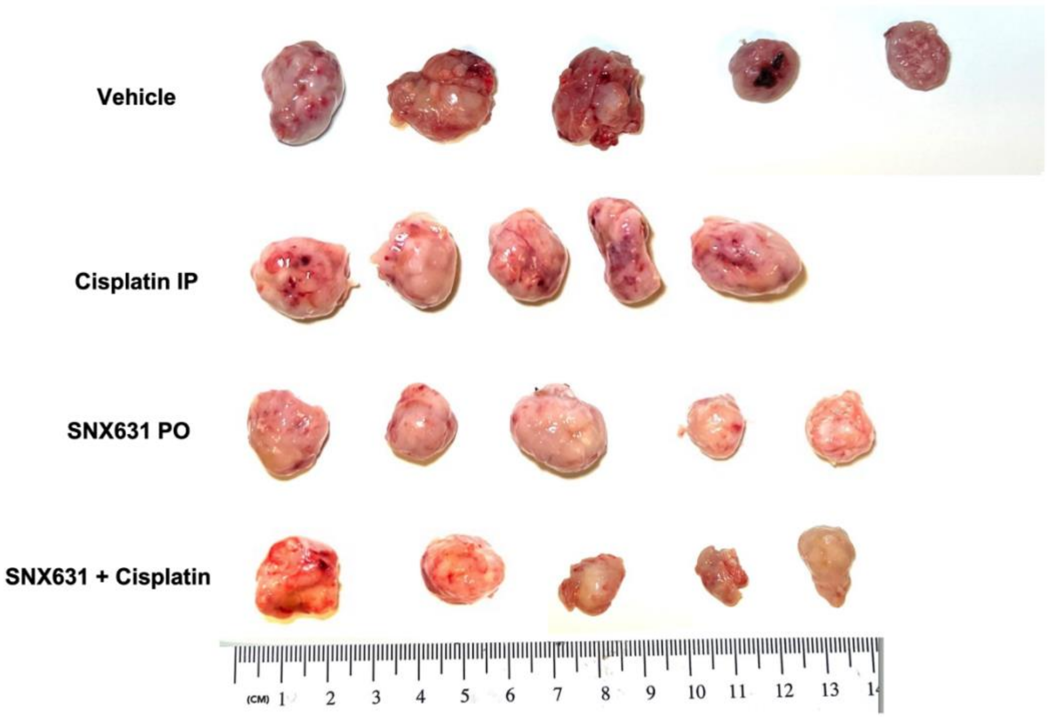

Supp Figure S5: Representative images of subcutaneously injected tumors from the four treatment groups as indicated collected at the endpoint: vehicle, cisplatin, SNX631 oral (PO), and SNX631 + cisplatin.

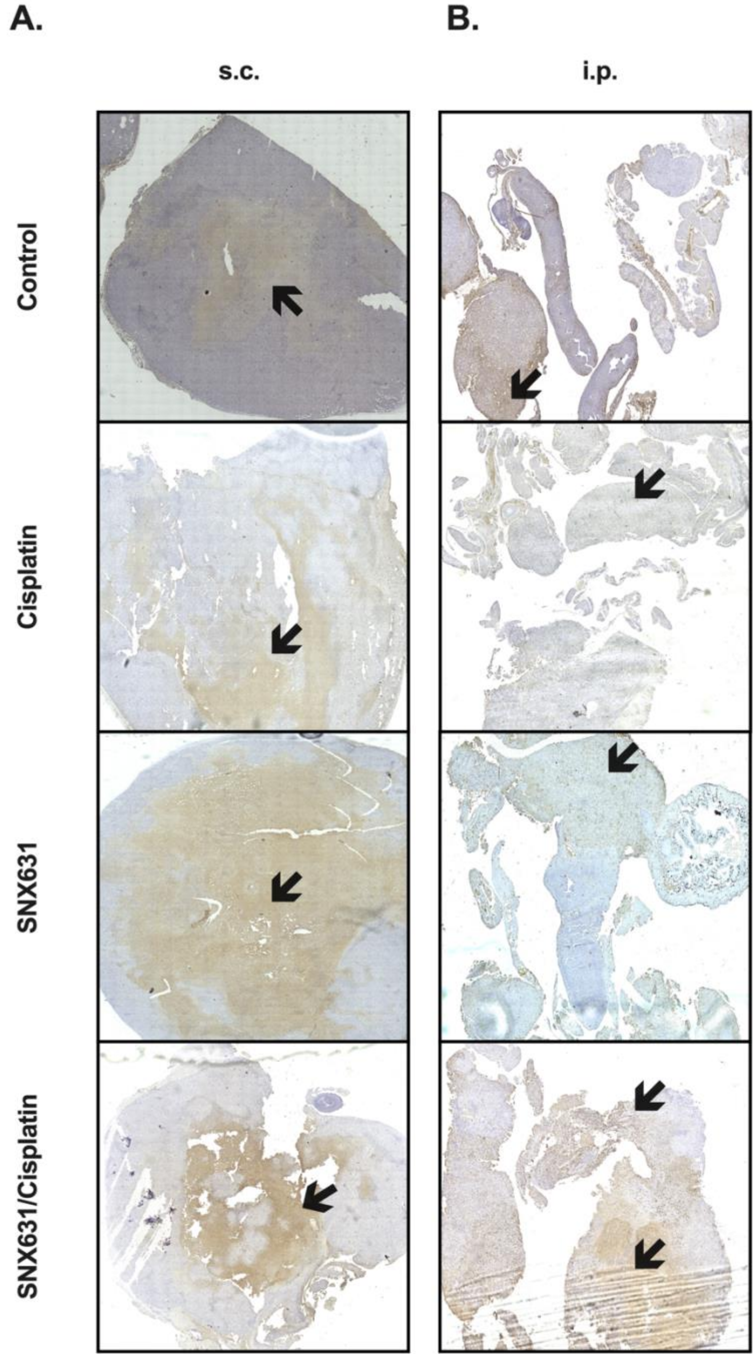

Supp Figure S6: Full tumor pictures from Figure 4. Black arrows indicate tumor necrosis.

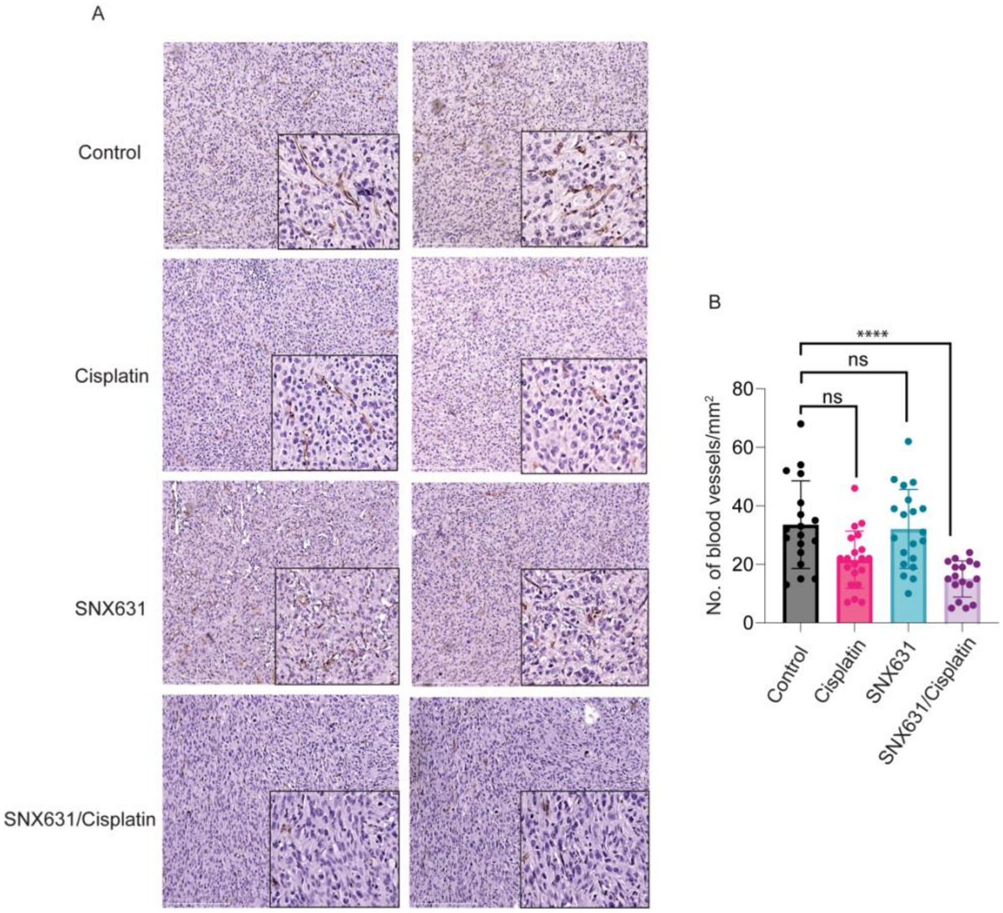

Supp Figure S7. A. Representative Immunohistochemistry images of subcutaneous tumor sections, stained with anti-CD31. **B.** Graph showing blood vessel counts from each group: Control, Cisplatin, SNX631, and SNX631/Cisplatin. A total of ten fields/image (x20) per tumor were counted, (Mean ± SEM, (n=2 independent tumors) ****p<0.0001, unpaired t-test.

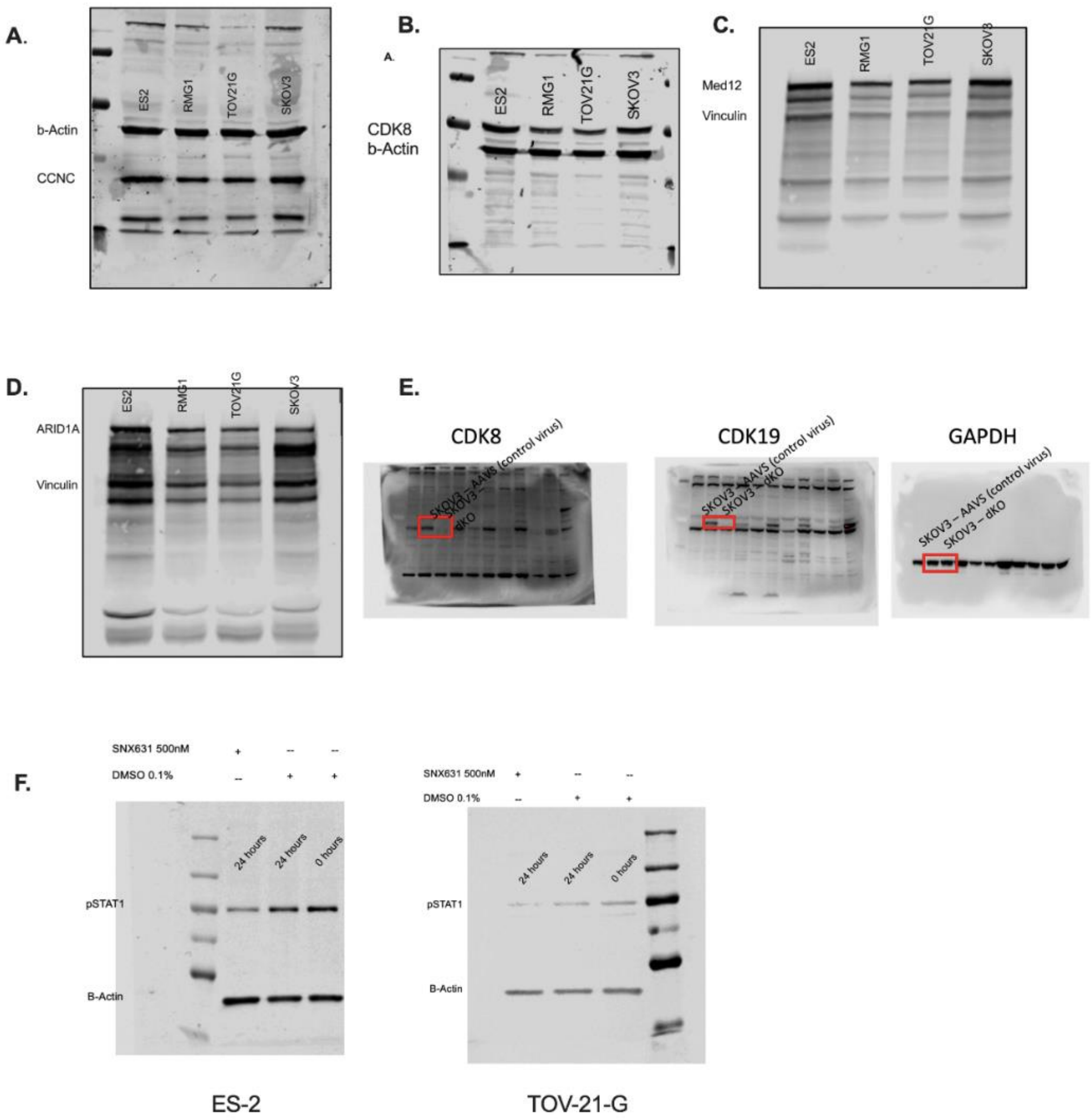

Supp Figure S8: Uncropped blots from. (A–D) Figure 1; (E) Figure 2; (E,F) Supp figure S1A
